# The INfoMATAS project: Methods for assessing cerebral autoregulation in stroke

**DOI:** 10.1177/0271678X211029049

**Published:** 2021-07-19

**Authors:** David M Simpson, Stephen J Payne, Ronney B Panerai

**Affiliations:** 1 Institute of Sound and Vibration Research, University of Southampton, Southampton, UK; 2 Institute of Biomedical Engineering, Department of Engineering Science, University of Oxford, Oxford, UK; 3 Department of Cardiovascular Sciences, Leicester Royal Infirmary, Leicester, UK

**Keywords:** Cerebral autoregulation, stroke, cerebral blood flow, hemodynamic regulation, signal processing

## Abstract

Cerebral autoregulation refers to the physiological mechanism that aims to maintain blood flow to the brain approximately constant when blood pressure changes. Impairment of this protective mechanism has been linked to a number of serious clinical conditions, including carotid stenosis, head trauma, subarachnoid haemorrhage and stroke. While the concept and experimental evidence is well established, methods for the assessment of autoregulation in individual patients remains an open challenge, with no gold-standard having emerged. In the current review paper, we will outline some of the basic concepts of autoregulation, as a foundation for experimental protocols and signal analysis methods used to extract indexes of cerebral autoregulation. Measurement methods for blood flow and pressure are discussed, followed by an outline of signal pre-processing steps. An outline of the data analysis methods is then provided, linking the different approaches through their underlying principles and rationale. The methods cover correlation based approaches (e.g. Mx) through Transfer Function Analysis to non-linear, multivariate and time-variant approaches. Challenges in choosing which method may be ‘best’ and some directions for ongoing and future research conclude this work.

## Introduction

Cerebral Autoregulation (CA) refers to the ability of the brain to maintain approximately constant blood flow in response to changes in blood pressure. Assessing CA remains a major challenge, with multiple methods and approaches having been described in the literature. Given the still limited understanding of the physiological processes and the construct of autoregulation, defining an index that seeks to quantify how well autoregulation is functioning in an individual subject is not a simple task. It is thus not surprising that still no gold-standard approach for assessing autoregulation has emerged, neither for research nor for clinical use. The current paper is the third in a series of papers published by the Cerebral Autoregulation Research Network (CARNET, www.car-net.org) to support collaborative research in CA. In line with this goal, in 2016 a new project entitled INFOMATAS (Identifying New targets FOr Management And Therapy in Acute Stroke) was launched with the objective of moving the extensive research in CA towards enhanced clinical benefit for patients after stroke. The first two papers outline the underlying physiology and integrative physiological assessment of cerebral hemodynamics in acute stroke, and this paper focuses on the assessment of cerebral autoregulation from physiological measurements. Subsequent papers focus on trials and interventions in stroke.

**Figure 1.**
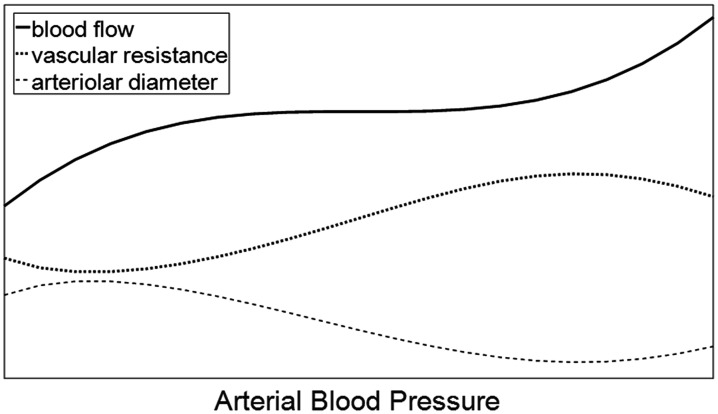
Static Autoregulation curve, redrawn after Paulson et al.^1^ The region where blood flow remains approximately constant inspite of increasing pressure shows active autoregulation, achieved by progressively increasing vascular resistance. This breaks down at the lower and upper limits of the autoregulatory range.

**Figure 2.**
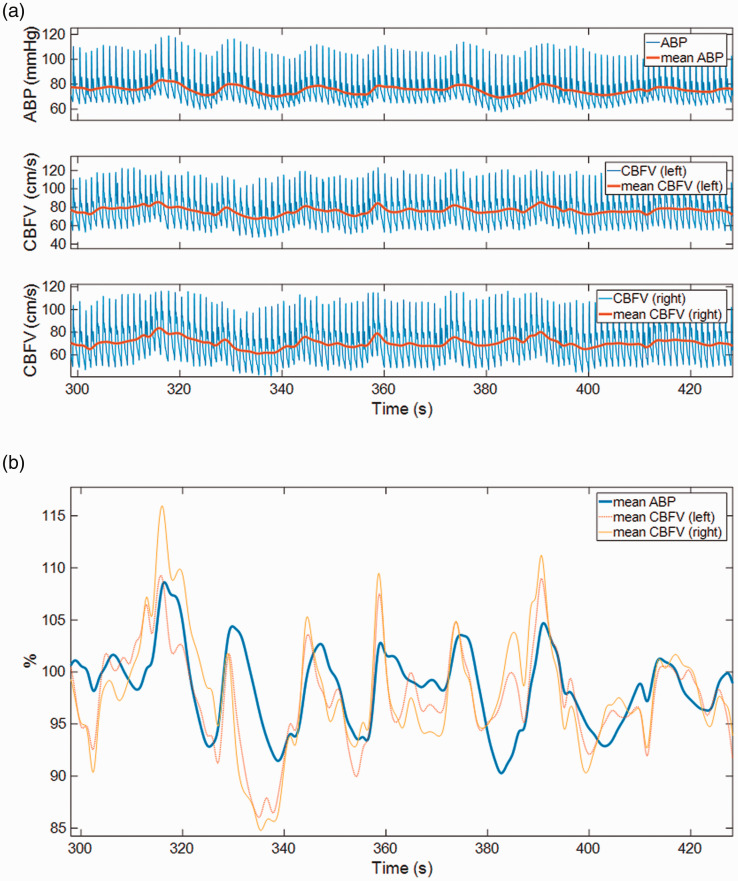
An example of arterial blood pressure (ABP), Cerebral Blood Flow Velocity (CBFV) showing spontaneous variations during rest. A) Raw signals as recorded, together with beat-averaged (and interpolated) mean signals. B) Normalized ABP and CBFV signals (shown as % relative to mean values). The left-shift of the CBFV with respect to ABP is clearly evident, with peaks in CBFV generally occurring before the peaks in ABP, and CBFV returning to baseline generally more quickly than ABP does. This illustrates the effect of dynamic autoregulation.

Two distinct aspects of autoregulation have been extensively studied in the literature: *static* and *dynamic* autoregulation. Static cerebral autoregulation^1,2^ (sCA) refers to changes in blood flow in response to changes in blood pressure in the ‘steady state’ (i.e. where mean blood pressure changes to a new level and is held there for minutes or hours) (Figure 1). In dynamic cerebral autoregulation,^2^ the transient responses of blood flow to changes in blood pressure are studied (Figure 2), with changes occurring within a few seconds being considered. This dCA can be observed during induced fluctuations in blood pressure (e.g. using a pressurised thigh cuff and its release) or with the small fluctuations that occur spontaneously in arterial blood pressure (BP) and cerebral blood flow (CBF) (as shown in Figure 2). Dynamic CA has become the focus of most current research and clinical assessment of CA in stroke,^3^ and will thus be the main focus of this review.

**Figure 3.**
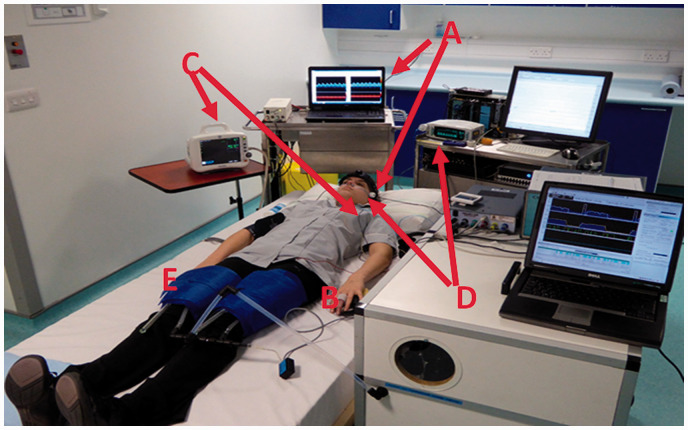
Experimental set-up showing TCD (A), Finapres (B), ECG (C), capnograph (nasal prongs) (D), and inflatable thigh-cuffs (E) (photograph taken with permission at Southampton General Hospital). The figure also shows the dedicated device for computer-controlled inflation/deflation of thigh cuffs and corresponding laptop for controlling and monitoring this process.^78,79^

Figure 3 shows an example of a (fairly typical) experimental set-up for assessing dCA, with a Finometer (Finapres Medical Systems, The Netherlands) for measuring BP, bilateral transcranial Doppler Ultrasound (TCD) probes (Dopplerbox, DWL, GmbH, Singen, Germany), nasal prongs for measuring end-tidal CO_2_ (as a non-invasive measure of PaCO_2_; Capnocheck®, Smith Medical, Ashford, UK), ECG leads for measuring heart-rate (ECG100C BIOPAC Systems Inc, Goleta, CA, USA), as well as (for this particular series of experiments) cuffs around the legs to induce transient changes in BP (developed at Leicester Royal Infirmary, UK). Quantifying the relationship between BP and CBF (or more commonly CBF velocity – CBFV – as obtained from TCD) is by definition at the core of all measures of autoregulation. We will give a brief description of methods currently used in the literature, including protocols for collecting and pre-processing signals, mathematical models that underpin analysis and method for estimating indices that quantify autoregulation.

All methods considered in this work^4^ are based on simultaneous recordings of BP and CBF (and sometimes more variables) taken under a range of different protocols, usually for periods from a few minutes to a few hours. There is also a substantial body of work that uses an alternative concept of autoregulation based on the relationship between blood pressure and intracranial pressure (ICP),^4^ focused on the PRx index. Given that this index requires invasive recordings of ICP, which are not usually available in patients after a stroke, this review will not expand on this approach. Other alternatives use tissue oxygenation level and the TOx index,^5^ based on Near Infrared Spectroscopy (NIRS), or a surrogate measure of intracranial volume^6^ and a Volume Reactivity Index (VRx), but these have also not been widely adopted in stroke studies.^7^ We will focus primarily on data collection methods for blood flow that can be used at the bedside, as these are most appropriate for patients following a stroke, but for the sake of completeness, we will also mention some alternatives, including MRI. The most direct ways of estimating CA is to impose changes in blood pressure and autoregulation can then be immediately observed (and quantified by simple parameters) from the responses in blood flow; however, some of these protocols are only suitable for relatively fit volunteers and only a few are suitable for vulnerable patients after a stroke. More complex signal analysis methods are required when evidence of autoregulation is less obvious, for example during smaller fluctuations in blood pressure, including spontaneous variability in BP. Two main approaches have been considered in such cases, quantifying 1) how strong the relationship between blood flow and blood pressure is – with a tighter linear coupling (higher correlation and coherence – see later sections) when autoregulation is impaired^8,9^ or 2) specific features of that relationship (e.g. gain or phase – see later sections). The latter are usually based on some predetermined mathematical structure to model that relationship (e.g. linear,^10^ non-linear,^11^ wavelets, Wiener Laguerre, empirical modes,^12^ etc.) and a range of mathematical and computational tools to estimate parameters from the signals. The current review is focused on CA assessments carried out at specific times (point measurements), rather than continuous monitoring of CA. The latter is becoming a common practice in patients after trauma,^13,14^ but currently not in stroke.

The main goal of the current paper is to shed some light on this complex topic. It aims to assist conceptual understanding of method and approaches, rather than giving detailed technical information. We hope it will help readers to better understand the literature and to make informed choices in developing their own research.

## Protocols for assessment of CBF autoregulation

Assessment of CA requires simultaneous measurements of CBF and BP. Ideally, the arterial partial pressure of carbon dioxide (PaCO_2_) should also be recorded given its very strong influence on CBF.^15^ Most studies derive these quantities using non-invasive techniques, such as transcranial Doppler ultrasound (CBF velocity or CBFV), arterial volume clamping of the digital artery (BP) (e.g. Finapres) and end-tidal CO_2_ (etCO_2_, as an estimate of arterial CO_2_ levels, PaCO_2_).^16^ Near Infrared Spectroscopy (NIRS) has been used as an alternative to TCD in some studies, including for stroke^5,7,17^ but TCD has remained the predominant method and will therefore be the main focus in this paper. There is some concern with NIRS regarding the impact extracranial (scalp) blood flow may have on measurements, and the susceptibility to movement artefacts.^7^ It may be noted that most of the modelling techniques described in this paper can be applied to both TCD and NIRS. Despite many years of research, the optimal protocols for studying CA, either as a static (sCA) or dynamic (dCA) phenomenon remain undetermined and the advantages/disadvantages of different approaches continue to be hotly debated.^18,19^ In this section we discuss the different protocols that have been proposed, with an emphasis on their suitability for assessment of CA in patients with ischemic or haemorrhagic stroke. Greater detail on experimental methods are provided in a companion paper (‘Protocols for assessment of CBF autoregulation’ section) in this series.

### Static CA

Despite being superseded by dCA, sCA is still reported in the literature and it is important to call attention to its limitations, in most cases a direct result of the protocols adopted to induce changes in mean BP (MAP) that should be maintained for several minutes. Protocols based on only two levels of MAP have been criticised due to the large experimental and numerical errors involved.^2^ Multiple levels of MAP should be observed in order to assess whether the slope of the mean CBFV-MAP relationship should be considered ‘flat’ (i.e. active sCA) or not.^20^ For this purpose, drugs inducing either hypertension (e.g. phenylephrine) or hypotension (e.g. nitroprusside) have been used.^1,21,22^ In addition to the invasiveness of the pharmacological manipulation, an additional limitation is the difficulty to control the stability and duration of the MAP plateau levels achieved using either a bolus or continuous infusion of these drugs.^21^

Other approaches to induce changes in MAP have been tilting or changes in circulatory blood volume.^23^ Although these protocols have the advantage of avoiding the use of pharmacological agents, it is still difficult to control the amplitude of the resulting changes in MAP, as well as its stability and duration. Moreover, both manoeuvres can induce autonomic nervous system activation that might interfere with the resulting changes in CBF. In patients with ischemic or haemorrhagic stroke, therapeutic changes in mean BP might give the opportunity to record parallel changes in CBF to obtain estimates of sCA, but otherwise inducing changes in BP is not recommended.

In addition to interventions to induce changes in MAP, sCA has also been assessed, within the framework of linear regression analysis, using spontaneous fluctuations of MAP. This approach would be better labelled as ‘quasi-static’ since in most cases no information can be obtained about the speed of the response. The Mx and Mxa indices are good examples of this approach.^8^ Despite the advantage of being non-interventional, a major limitation of using spontaneous fluctuations is the lack of control over the range of MAP values achieved which can affect the significance of results derived by linear regression analysis.^20^

### Dynamic CA

Assessment of dynamic CA relates the transient response of CBF to relatively rapid changes in BP, ideally taking place within 1–5 s. As listed in Table 1, several different protocols have been proposed to induce a single change or multiple changes in BP that can be combined with different analytical techniques (see below) to derive parameters reflecting the efficiency of dCA. On the other hand, spontaneous fluctuations in BP have also been extensively used for this purpose since the natural variability in BP usually contains beat-to-beat changes that can be regarded as sufficient to stimulate a dynamic CA response. The advantages and limitations of either approach (spontaneous vs induced) have received considerable attention.^18,19^ In summary, spontaneous fluctuations afford assessments at rest without any disturbances of physiological conditions, thus being ideal for clinical applications, particularly in patients following a stroke or otherwise critically ill, but the relatively small amplitude of BP fluctuations lead to less reliable estimates of dCA parameters. Induced changes in BP, on the other hand, lead to better signal-to-noise ratios and challenge CA more strongly, given the larger excursion of BP, but the corresponding effects on the underlying physiological conditions can generate additional ‘inputs’ to CBF signal thus distorting dCA estimates.^18^ Moreover, many of these protocols are not suitable for stroke patients, either due to safety concerns or limitations in the patients’ mental or physical ability to follow the protocol.

**Table 1.** Main characteristics of protocols for assessment of dynamic CA. Note that this list is not exhaustive, and that the use of the method in the literature (* rating) is based on subjective assessment by the authors (given the complexity of the methods and combinations used).

Method	Principle	Subject cooperation required?	Test duration (min)	Main limitations	Literature^a^	Key references
Spontaneous fluctuations	Rest	N	5	Reduced BP variability	*****	^10,44^
Thigh cuff release	Bilateral leg compression	N	1	Discomfort (pain), SA, SM	***	
Head up tilt	Induced hypotension	N	5	Equipment costs, SM	**	
Fixed breathing	6 breaths/min	Y	5	Hypocapnia	**	
Sit-to-stand	Repeated manoeuvre	Y	5	Exercise	**	^83^
Squat-to-stand	Repeated at 0.05 or 0.10 Hz	Y	5	Exercise	**	
Hand grip	3 min submaximal exercise	Y	5	SA, exercise, hyperventilation	**	^84^
Leg raising	Repeated passive movement	N	5	Exercise (when performed by subject)	*	
Valsalva manoeuvre	Forced expiration	Y	1	ANS activation, intrathoracic pressure rise, SM	*	
Carotid artery compression	Finger compression for 5 s	N	1	Carotid artery disease, SM	*	
Random repetitive thigh cuff compression	Multiple inflation/deflation for 5 min	N	5	Equipment costs, SA	*	^82^
						
Rapid head elevation	Repeated 0° – 30° - 0° head position 4x in 60 s	N	5	Light discomfort	*	
Cold pressor test	Hand in ice water	N	1	Pain, SA, SM	*	^84^
Lower body negative pressure	Pressure reduction by suction from waist down	N	5	Discomfort, SA	*	^84,85^

SA: sympathetic activation; SM: single measurement; ANS: autonomic nervous system.

^a^Approximate relative number of references found in the literature, subjectively estimated by the authors, based on their experience of the literature^16^. For spontaneous fluctuations (*****) this number is of the order of 300–500 studies.

Choosing which protocol to adopt is not straightforward, unless there is an obvious association with the research question being addressed, such as the study of dCA during exercise, or clinical studies where patients cannot tolerate any protocols apart from spontaneous fluctuations in BP. Given the lack of reference values (‘gold standard’) for dCA parameters, inter-method comparisons cannot provide a definitive answer as to which protocols should be preferred; they can provide complementary information, such as reproducibility and feasibility in different groups of individuals. Ultimately, only comparative studies of diagnostic/prognostic performance might indicate the relative superiority of some protocols in particular groups of patients. In the absence of this information, some characteristics of different protocols are worth noting. First, the amount of information available in the literature is very uneven, as indicated in Table 1, with some of the protocols being used in only a handful of studies. For obvious reasons, the limitations of the different protocols need close scrutiny. Protocols that yield continuous data, mainly due to repetitive manoeuvres (sit-to-stand, squat-stand, random repetitive thigh cuff manoeuvres, fixed breathing), can be analysed by transfer function analysis (TFA), assuming that the data are stationary (see ‘Data preparation’ section 3). However, for manoeuvres inducing a single transient change in BP (single thigh cuff deflation, cold stress test, carotid artery occlusion, sit-to-stand manoeuvre etc.), other analytical techniques need to be employed and these are much less well-established than TFA.

MAP is given by the product of peripheral vascular resistance (Rp) and cardiac output (CO). Since CO in turn is the product of stroke volume (SV) and heart rate (HR), MAP= (SV.HR).Rp. This relationship is useful to highlight the fact that it is not possible to induce changes in MAP without affecting one or more of these parameters, which may lead to confounding when attempting to assess changes in vascular resistance or CBF in response to changes in MAP. For the protocols where ‘exercise’ is given as a limitation in Table 1, one can expect all three parameters (SV, HR, Rp) to be affected, with the involvement of the sympathetic nervous system. Many other protocols also stimulate sympathetic nervous activity (SA in Table 1), either as the main pathway to MAP change (e.g. cold stress test), or as a response to the primary physiological changes induced (e.g. lower body negative pressure) or pain (e.g. thigh cuff manoeuvre). On the other hand, some manoeuvres are more specific. For example, leg elevation increases MAP due to increases in SV resulting from increased venous return. The single thigh cuff manoeuvre reduces Rp due to hyperaemia downstream to the cuffs,^24^ but when looked into in more detail, it also involves changes in PaCO_2_ and HR.^25,26^

As highlighted by a recent review from Xiong et al.,^17^ both ischemic and haemorrhagic stroke present a particular challenge for assessment of CA. As in any clinical studies, the first consideration is the safety implications of different protocols, followed by whether patient cooperation is needed or not (Table 1). Although some protocols in Table 1 that require cooperation could be performed by many stroke patients (e.g. periodic breathing), unless the manoeuvre can be used in all patients, there is the risk that patient recruitment, already a major challenge in these studies, might be further jeopardised. Until further evidence is provided that demonstrates the superiority with regard to feasibility, safety and diagnostic/prognostic accuracy of a specific protocol for inducing BP changes in stroke patients, our recommendation is the use of spontaneous fluctuations, associated with TFA.^10^

## Data preparation

Visual inspection is required to select good quality data free from major artefacts. Pre-processing to further reduce noise in the signals, followed by the extraction of beat-averaged signals is usually also required. These steps will be outlined in the current section. For CBF, only illustrations with CBFV from TCD signals will be shown, as these are most commonly used. Further details have been presented in Claassen et al.^10^

**Figure 4.**
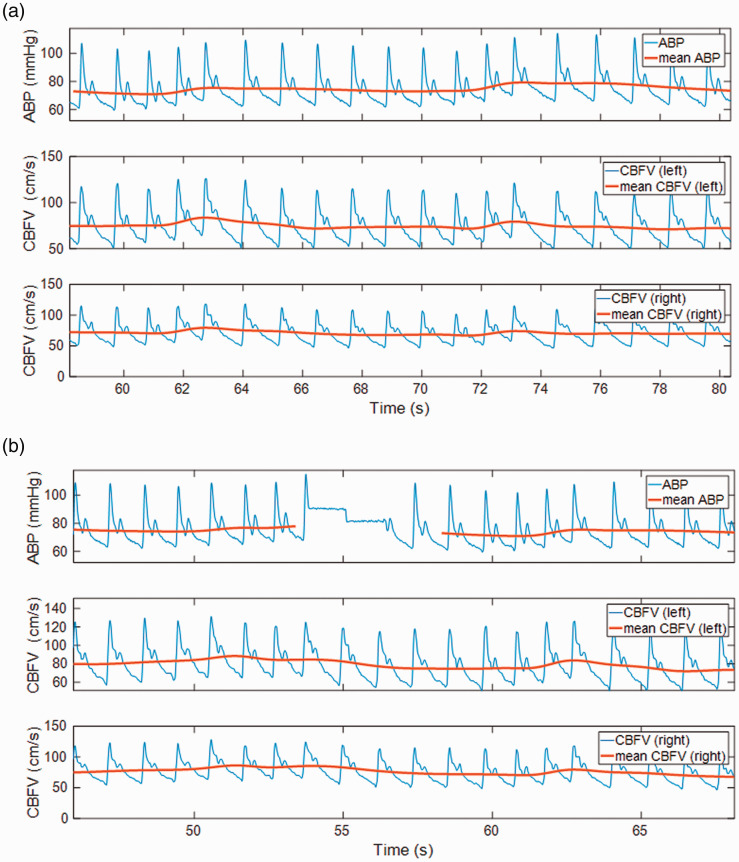

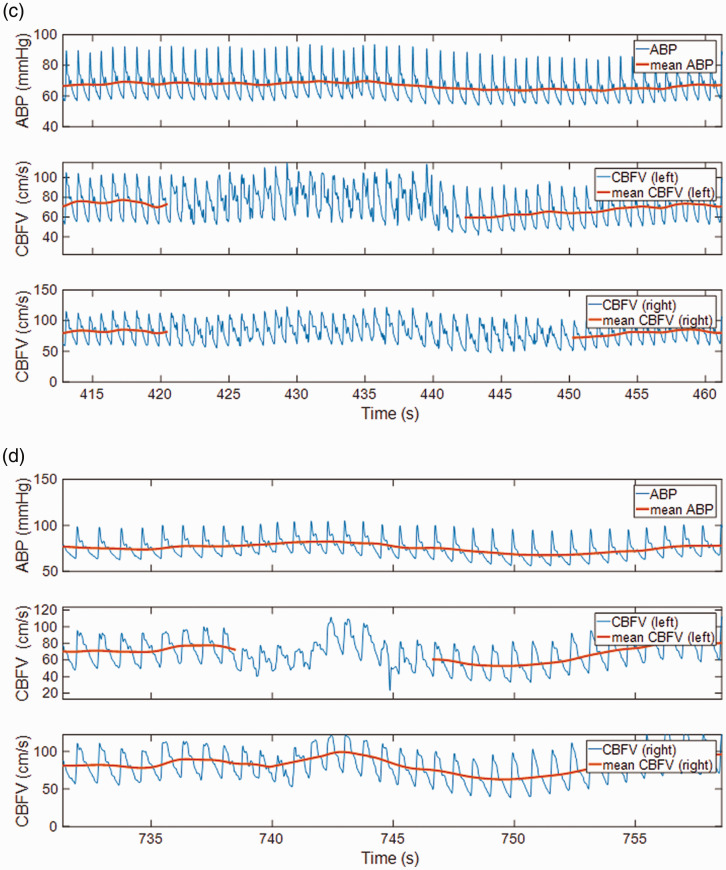
Examples of signals showing (a) good quality, (b) typical artefacts in BP, (c) and (d) typical artefacts in CBFV. The signal segments deemed inadequate for further analysis are indicated as gaps in the mean signals (red, bold). It may be noted that the CBFV signals in D are not of high quality throughout, and on the right side, it is questionable if the beats around 740 seconds should be included or not. This illustrates the challenge of compromise often required when ‘editing’ (selecting) segments of signal for further analysis.

Visual inspection should be carried out on the raw CBFV and BP signals, prior to filtering and beat-averaging (see Figure 2(a)), as pre-processing steps can hide artefacts. Good quality signals for CBFV and BP should show clear pulse-waves that are quite repeatable between beats, as shown in Figure 4. A clearly noted dicrotic notch in the downstroke after systole is a further indication of good quality (though this may not always be clearly visible in data that is otherwise acceptable). Figure 4 also shows some examples of typical artefacts in recorded signals. The most common artefacts in the TCD signal are due to relative movement between participant and probe, and are typically observed as a decrease in pulsatility, large spikes, or a more noisy (random) appearance of the signal. A general decrease in amplitude is also often seen to coincide with this. A comparison between changes in BP and CBFV can be helpful to assess if ‘surprising’ changes in the signals are physiological or not. Another common artefact of TCD is irregular spikes in the signals, arising from poor choice of thresholds (or changing signal strength) for the ‘maximum frequency envelope’ set on the Doppler device. The resulting artefacts can be reduced by median filtering^27^ or interpolation^25^ (or both), provided that they only occur sporadically.

Common artefacts of BP are drift (very common in finger plethysmographic devices) and a loss of pulsatility. Self-calibration (‘physiocal’), an automatic feature of some of these devices, leads to a typical artefact consisting of a series of steps in the signal (see Figure 4(b)). If possible, these should be avoided by switching off the self-calibration mode in short recordings. If present, they should be removed by interpolation when editing the data, provided that the segments eliminated are no longer than a few seconds in duration and do not occur too frequently.^28^

Short segments of artefacts in TCD or BP, up to about 3 beats long, can be removed by replacing them with (linear) interpolation between the good quality data before and after the artefact.^28^ Sporadic ectopic beats do not need to be removed, as they provide a physiological challenge to autoregulation.^29^

In order to further remove high-frequency artefacts and noise from the BP and CBF signals, low-pass filters, typically cutting off at 20 Hz, are applied in the next step. For dCA, the mean values of the signals are of no interest but can be large (compared to the fluctuations which are of interest), and are prone to drift (especially for non-invasive BP). Therefore mean values should be subtracted out of the signals, prior to analysis (and prior to filtering).^30^ Detrending recordings with the removal of the underlying linear trend is sometimes performed, but any more complicated detrending is usually not justified. Given that dCA is predominantly a low-frequency phenomenon, below approximately 0.2 Hz^10,31^ and especially at the very low frequencies (below approximately 0.05 Hz), higher frequency fluctuations (especially those due to heart-rate) should be removed prior to the assessment of CA. This can be achieved by averaging across each heartbeat (to calculate the beat-average value) of BP and CBFV (see Figures 2 and 4). The beats are ideally detected from the sharp R-wave in the ECG signal but can also be found from the pulse-foot, steepest gradient or peak of the BP signal. The samples thus obtained, one for each heartbeat, then need to be interpolated (for example linearly or by cubic splines) to obtain a regularly sampled signal. Sampling rates used after this step are typically 10 Hz or lower.^10^ An alternative approach is to low-pass filter the raw signals with a cut-off frequency of around 0.2 Hz, but this can lead to any artefacts in the signal spreading more widely over time (more than to the next beat). These beat-averaged (or low-pass filtered) signals (shown in bold lines in Figures 2 and 4) will be denoted by *p(t)* and *v(t)* (or for simplicity, *p* and *v*) for BP and CBFV respectively and form the input to the analytical methods described in the remainder of this paper.

In some cases it is desirable to normalize these signals by their mean value, and express them as % change relative to their mean.^10^ This is particularly true for the CBFV, since it gives velocity (in cm/s) rather than volume flow (in ml/s); but the two are proportional when there is no change in diameter of the insonated artery. In the absence of CA and with a rigid vascular system, one might then expect a given percentage change in *p* to be accompanied by an equal percentage change in *v*. An additional concern is that the pressure at the outflow of the brain is not zero, but has been variously taken to be venous pressure,^32^ intracranial pressure^8^ or critical closing pressure (the pressure at which veins or venules collapse).^33^ It is therefore often recommended that perfusion pressure (the pressure drop across the cerebral circulation) be used when considering the relationship between *p* and *v.*^33–35^ BP is of course just an approximation to this and will not reflect the true cerebral perfusion pressure in the presence of intracranial hypertension as observed in many conditions such as severe head injury.^8^

Given the importance of hyper- and hypocapnia on blood flow and CA, estimates of PaCO_2_ levels should be obtained when possible. The most common estimator are the Respiratory end-tidal CO2 levels (etCO_2_) from capnography (see ‘Protocols for assessment of CBF autoregulation’ section). These are usually obtained as a continuous signal by linearly interpolating between the maximum (end-tidal) CO_2_ values from consecutive respiratory cycles – and again this signal needs then to be interpolated to achieve a sampling rate compatible with that of *p* and *v*. Further details on suggested signal pre-processing can be found in literature.^10^ The output of these processing steps then feed into the analysis and extraction of parameters that quantify CA.

## Single input, time-invariant, linear modelling techniques for assessment of CBF regulation

In this section we will progressively build up the different linear approaches to assessing dCA. Table 2 provides a list of some of the more commonly-used indices of autoregulation, and the next paragraphs aim to provide a conceptual framework that links these methods.

**Table 2.** Main characteristics of some (linear) indexes used in dCA assessment. Note that this list is not exhaustive, and that the use of the method in the literature (* rating) is based on subjective assessment by the authors (given the complexity of the methods and combinations used).

Index	Principle	Experimental protocol	Strengths	Weakness	Literature#	Key references
Rate of Regulation (RoR; ΔARi)	Rate of change in CBFV normalized by change in BP	Transient change in BP, specifically thigh-cuffs	Simple interpretation; can be used on estimated step-responses	Can only be used with clear transients in BP	**	^24,39^
Mx/Mxa (Sx)	Correlation between CBFV (mean or systolic) and cerebral perfusion pressure (CPP) – requiring ICP or BP	Rest with spontaneous variations	Widely used in literature; Extensively tested in clinic; insensitive to signal scaling/normalization	Correlation biased downwards by noise; requires typically 30 minutes of recording; some measures require ICP	***	^8,17,36^
ARI (ARMA-ARI)	Fitting pre-defined set of linear filters	Thigh-cuff; Rest with spontaneous variations	Simple interpretation	Set of models is not optimized	***	
Phase (in very low, low and high frequency bands)	TFA (parametric or non-parametric)	Rest with spontaneous variations; repeated transients	Insensitive to signal scaling/normalization; Extensively used for low frequency band	TFA can give aberrant results	****	^10,40,41,86^
Gain (in very low, low and high frequency bands)	TFA (parametric or non-parametric)	Rest with spontaneous variations; repeated transients		TFA can give aberrant results; sensitive to signal scaling	****	^10^
Coherence (in very low, low and high frequency bands)	TFA (parametric or non-parametric)	Rest with spontaneous variations; repeated transients		TFA can give aberrant results; coherence biased downwards by noise	**** (mostly for the purpose of checking robustness)	^10,44^

In its simplest form, one may approximate the linear relationship between flow and pressure as

(1) v[i]=h.p[i]+e[i]

where *v[i]* is CBF(V), *p[i]* is BP (with *p* and *v* recorded simultaneously), *h* is the gain or gradient and *i* refers to the *i*th sample in the recording (i.e. equivalent to time, but measured in samples rather than seconds). Note that here both *p* and *v* are assumed to have a mean value of zero (after pre-processing), and usually represent beat-averaged signals, or some smoothed (filtered) version of these. *e[i]* is called the error or residual and represents all the variations in *v* that cannot be explained by *p* with this simple model, and thus includes noise in measurements, delayed interactions between *p* and *v*, non-linear or time-varying behaviours in CA, additional inputs (such as PaCO_2_ or ICP) or other related phenomena. In the case of normalized signals (expressed as % change), *h* < 1 would indicate some active autoregulation, as the change in *v* is less than that in *p*. When flow and pressure measurements are made repeatedly over relatively long time-intervals with significant changes in pressure between them (e.g. due to drug infusions), *h* represents static autoregulation (sCA) (the gradient of the static autoregulation curve). It may also be noted that in this model *h* is invariant over time as *h* is assumed to be a constant over the full length of the recording (see later section on time-variant methods).

This model also underlies the set of CA indices proposed by Czosnyka et al. (including Mx or Mxa),^8,17^ where repeated measurements are obtained by averaging continuous recordings of BP and CBFV over approximately 5 second long contiguous intervals, and then relating *v* and *p* over typically around 40 such averages. The smoothing function used in their calculation, with samples taken every 5 seconds, means that only quite low frequency variations in the signal (below 0.1 Hz) can be analysed. Rather than calculating gain however, they calculate the correlation coefficient, i.e. a measure of how well this linear model fits the data (or how small *e[i]* is, on average). The rationale is that when CA is absent, the relationship between pressure and flow is passive and thus linear, and correlation should be high. In the presence of an active autoregulation, this linear relationship breaks down and the correlation coefficient will decrease. One might question this approach by considering that the correlation coefficient will also decrease if the data are noisy or contain artefacts: this approach will thus only be robust when data quality is relatively high, for example in heavily sedated or unconscious patients. Mx or Mxa have also been used in stroke,^17^ but have not become the predominant approach. One debate is whether Cerebral Perfusion Pressure (CPP=BP – ICP or CPP=BP – CrCP, where CrCP is the critical closing pressure) should be used (leading to Mx), or whether BP is a ‘good enough’ approximation^36^ (leading to Mxa). It seems clear that CPP would be ideal, but this may require ICP to be monitored, which is only possible in some patient groups. An alternative is to estimate CrCP from the recordings of *v* and *p.*^33^ This estimation of CrCP has however so far not been very widely adopted^36^ but investigations continue.^37^

The next more sophisticated model includes ‘memory’, i.e. the current measure of blood flow is affected not only by the current value (sample) of blood pressure, but also by a number of preceding samples.

(2) $$v\left[i\right]=\sum_{k=0}^{K}h\left[k\right]p[i-k]+e[i]$$

*h[i]* may now be considered as a set of ‘gains’ and represents the ‘impulse response’ of the system. This equation represents a linear filter. *K* is the length of the impulse response (in samples; often called the ‘order’ of the filter) and corresponds to the length of time over which a change in *p* is deemed to affect *v*. Typically this would correspond to somewhere between 5 and 15 seconds, with *h[i]* decaying towards zero over this period.

CA can then be assessed by first estimating *h[i]* from recordings of *v[i]* and *p[i]*. The ‘best-fit’ *h[i]* is obtained when *e[i]* is minimum, i.e. *v[i]* is explained as much as possible by *p[i]* and the unexplained component (*e[i]*) is thus minimized. Typically (for ease of computation), the mean of the square value of *e[i]* over the length of the recording is minimized (least-mean-square estimate). When *h[i]* has been estimated, it is possible to predict the response of the (mathematical) system to alternative input signals (*p*), such as an impulse, a step or sine waves at different frequencies. The former lead to the estimation of the impulse and step responses, respectively, much used in the literature.^38,39^ The latter leads to the frequency response.^2,10,40,41^ For active autoregulation, one would expect the step response to show a peak at the onset of the step and then a rapid decay towards zero, as autoregulation brings the flow back towards baseline (pre-step values). The analysis of the response in the CBFV following an impulse, step or other transient is called *time domain* analysis. When considering the response to sine or cosine-waves of different frequencies (using Fourier analysis), this is called *frequency domain* analysis.

**Figure 5.**
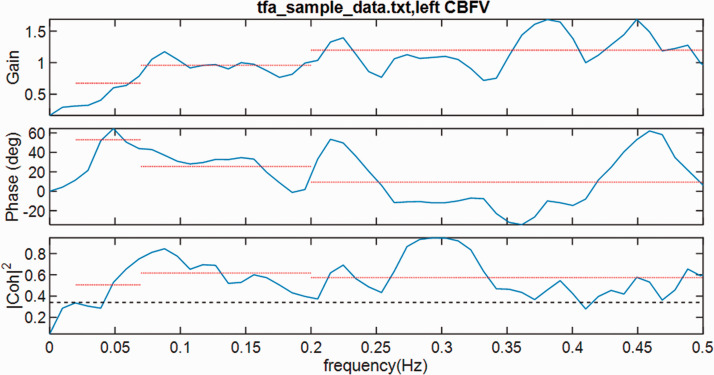
Transfer function analysis showing (from top to bottom), the gain, phase and coherence. The dotted lines indicate the average value over selected frequency bands (very low, low and high). The dashed line in the coherence plot indicates the critical value (95% confidence limit): if coherence falls below this level, then the corresponding frequencies should be excluded in calculating the average phase or gain. Figure generated from TFA_demo.m, in CARNET software at http://car-net.org/content/resources.

With the frequency response, for a unit-sized sinusoidal input at a given frequency, the amplitude of the output signal represents the gain (or amplitude frequency response), and the delay (or advancement) of the output relative to the input gives the phase frequency response (see Figure 5). A delay of one complete cycle of a sine wave corresponds to 360 degrees (or 2π rad). In the frequency response, the response of *v* to low-frequency fluctuations in *p* should be more strongly attenuated than that for high frequencies (as CA is less effective in response to fast changes in BP). This would imply that gain should increase with frequency, and hence resemble a high-pass filter effect. A low value of average gain in the low frequencies is indeed commonly used as an indicator of active dCA. In addition, one may expect that CA will suppress the increase in *v* as pressure rises, leading to the peak of *v* occurring before the peak in *p*, usually described as a phase lead or a positive value in phase at that frequency. This phenomenon (though not with a pure sine wave) can also be observed in the apparent left-shift of *v* relative to *p* in Figure 2. A large phase lead at low frequencies is thus used as an indicator of active CA.

The frequency response is usually obtained by applying the Fourier transform to the impulse response, rather than explicitly inputting sine waves into equation (2). Alternatively, and more commonly, the frequency response is obtained by ‘Transfer Function Analysis’ (TFA),^2,10^ with Fourier transforms applied to the signals rather than the impulse response. The mathematical model underlying TFA is very similar to that in equation (2). One difference is that the equation should be expanded with the sum becoming

(3) $$v\left[i\right]=\sum_{k=-\frac{N}{2}}^{\frac{N}{2}-1}h\left[k\right]p\left[i-k\right]+e[i]$$

where N is the length of the window (in samples) used in estimating the transfer function. This suggests that *v[i]* is associated with future values of *p[i]* as well as the past, as would occur if the interaction between *v[i]* and *p[i]* is mutual, rather than simply causal, with *p[i]* driving *v[i]*. Such apparent non-causal behaviour can however also arise from the smoothness of the signals.

Further details on recommended approaches to TFA for the study of CA are given in literature.^10^ We strongly recommend that for TFA, the procedures outlined in literature^10^ are followed, and for which Matlab® code has been provided at http://www.car-net.org/.

The basic model in equation (2) can furthermore be expanded as

(4) $$v\left[i\right]=\sum_{k=0}^{\infty }h\left[k\right]p\left[i-k\right]+e\left[i\right]$$

where now the ‘impulse response’ h[i] is infinite (the sum involves an infinite number of terms). Alternatively, with some constraints, this can be expressed through a finite sum by recursion,

(5) $$v\left[i\right]=\sum_{k=1}^{L}a\left[k\right]v\left[i-k\right]+\sum_{k=0}^{K}b\left[k\right]p\left[i-k\right]+e\left[i\right]$$ –

where *v[i]* is found from *p[i]* and now also previous values of *v[i]* (representing the recursive component), using parameters *a[k]* and *b[k]*. The ‘parametric’ estimate of the transfer function^42^ is based on finding these parameters as a first step in identifying the mathematical model linking *p* and *v*. This model is also the one used in the ARMA-ARI method.^38^ When all parameters *a[k]*=0, this simplifies to the finite impulse response system, as given in equation (2), with *h[k]*=*b[k]*. The parametric approach provides an alternative to the conventional TFA method^10^ that has shown some performance benefits.^43^

Following the same reasoning used in proposing correlation analysis above, one can also use frequency selective correlation (known as coherence) to assess dCA. There are, however, currently two somewhat contradictory interpretations in the literature. One line of thought is that (as with correlation), low coherence would suggest a non-linear system, and therefore CA is active.^44^ The second line of thought is that transfer function analysis should only be carried out when coherence is relatively high (e.g. statistically significant), otherwise estimates are unreliable.^10,30^ Putting these two lines together suggests the phase or gain should only be estimated when dCA is impaired (leading to high coherence). The assumptions in these interpretations are however open to debate: low coherence does not necessarily arise from active (non-linear) autoregulation, but could also come from other phenomena, including noisy signals or other time-varying inputs (such as PaCO_2_, metabolic activity, ICP fluctuations). Low coherence could thus reflect poor data, rather than active CA. Secondly, while autoregulation is clearly non-linear, a linear approximation (a high-pass filter) may come close, with the error (*e[i]*) dominated by the noise rather than non-linear effects. In that case the frequency response can provide a good indication of active autoregulation, provided that the coherence is not so low (typically a threshold of 0.5 is used – though this value is poorly supported by evidence^10^) so as to make the estimates unreliable. Alternative ways of estimating coherence using ‘wavelets’ rather than Fourier Transforms have also been discussed.^45–47^

The parametric model given above also encompasses the approach used in the ‘Autoregulation Index’ (ARI), as proposed by Tiecks et al.^22,38^ Here ten sets of fixed parameters (which can be used to find *a[k]* and *b[k]*) provide ten alternative impulse responses (and hence step responses or transfer functions) that go from absent autoregulation (passive response of CBFV to a step-change in BP; ARI = 0) to excellent autoregulation (ARI = 9). Rather than estimating *h[k]* (or *a[k]* and *b[k]*), one can therefore just chose which of these ten filters best fits the data (minimizing *e[i]*). The Tiecks model can thus be viewed as a constrained version of Transfer Function Analysis, where only a small number of parameter values (*a[k]* and *b[k]*) are tested.

The simple linear models discussed so far already point towards the challenge of selecting and interpreting indexes to quantify dCA. There is a plethora of parameters that have been extracted from these mathematical models, all deemed to reflect CA, and with a wide range of evidence that they do so. Some of these are summarized in Table 2 (with the table completed from parameters derived from more complex models discussed in later sections of this paper). It should be noted that agreement between different methods has not always been found to be strong,^36,48,49^ and sometimes quite weak. It should also be pointed out that implementation of different methods has not always led to identical results from different research groups, even when they thought they had used the same method.^50^

In addition to the more widely used parameters given in Table 2, there are a series of parameters extracted for example from linear models and the estimated step response (e.g.^39,43^). Visual inspection of these responses is also extensively used to confirm autoregulatory behaviours and validate results.^51^ Indeed, visual inspection of impulse, step and frequency responses is highly recommended in all of these analyses to avoid extracting indexes from aberrant responses. An automatic procedure based on critical values of coherence (in the 0.15–0.25 Hz range) and the normalised mean squared error has also been proposed for acceptance or rejection of the CBFV step response.^52^

## Alternative modelling approaches

In the previous section, we examined the use of single-input (BP or CPP), linear, time-invariant modelling techniques. We now turn our attention to alternative approaches that attempt to relax these assumptions about the model behaviour in the context of cerebral autoregulation. Such methods have been used in stroke, but currently are not very extensively applied.

### Multivariate models

Early studies into dynamic autoregulation clearly showed that the parameters recovered are dependent upon the levels of blood gases, in particular changes in CO_2_.^24,41,53^ Rather than consider the effect of CO_2_ on these parameters, some more recent studies have explicitly modelled the multivariate nature of cerebral autoregulation. In these models typically equations (2), (3), (4) or (5) above are extended to include other inputs and their parameters (gains or impulse responses). Multivariate transfer function analysis has shown a significantly higher multivariate coherence than univariate coherence in the frequency range below 0.04 Hz,^54^ with the multivariate gain also significantly higher than the univariate gain^9,55^ although effects in the low frequency range are smaller. It is now widely acknowledged that changes in PaCO_2_ should be taken into consideration when assessing cerebral autoregulation and multivariate analysis would be strongly recommended if PaCO_2_ (or etCO_2_) shows marked changes throughout a recording.

### Non-linear models

Linear models, such as those given in equations (1) to (4), view CBFV as a weighted sum of past and present (and future, in the case of equation (3)) samples of BP. In the sums there are no squared or cubed (or other non-linear) terms of BP, nor are there any ‘cross-terms’, such as *p[i-k]p[i-m]*. This makes them linear and greatly simplifies analysis. However, certain behaviours, such as responses to positive or negative steps in BP being asymmetrical or the saturation of autoregulatory responses under very large changes in BP (beyond the plateau), cannot be represented in these simple models. Non-linear terms need to be included to overcome these limitations of the linear models. The autoregulation process, by its definition, cannot be strictly linear. The question is whether the linear approximation and simplification is sufficient for the assessment of autoregulation in typical clinical and experimental scenarios (i.e. over a relatively small range of blood pressures). As indicated by the principle of parsimony, the simpler linear models generally have the advantage of being statistically more robust in estimation, when the approximation can be justified.

There have been a number of studies into the non-linearity of cerebral autoregulation (e.g.^56-59^), based on the evidence for this cited (e.g.^56-61^): in particular by the low values of coherence exhibited at very low frequencies. However, the representation of a non-linear system is dependent upon the method used to derive it, which makes both comparing results across studies and interpreting the results highly challenging. The additional complexity of a non-linear model also means that considerably more data are required to fit such a model, which can be a major limitation on the use of these methods. There is also the associated risk of ‘overfitting’ (fitting the model accurately to the specific data recorded, including the noise, but not modelling the inherent behaviour of the system robustly), which grows with the increasing complexity of the model, and the associated growing number of free parameters.

The first study into the non-linear behaviour of cerebral autoregulation,^56^ used the quadratic Volterra-Wiener kernel, which was calculated in addition to the linear kernel, although over-fitting of the data was found. This was extended by Mitsis et al.^57^ to include both a fast and a slow component to autoregulation (with the fast component being found to be substantially the larger of the two), using Laguerre-Volterra networks. The reader is referred to these papers for the mathematical details of these techniques; it should be noted that extracting variables from these models that relate to autoregulation is a non-trivial task. Other non-linear models that have been proposed include neural networks^58^ and Support Vector Machines.^59^

### Time-variant models

Equations (1) to (5) all assume that the model parameters (*h[k]*, *a[k]*, *b[k]* …) do not change over time, and thus they all represent time-invariant models. The most commonly used multivariate and non-linear models are also time invariant. Time variant models have been used to track changes in autoregulation over time, as well as to assess the temporal variability exhibited by autoregulation. The simplest way of doing this is to use a sliding window (normally rectangular) that passes across the time series and to analyse dCA in successive windows, using the standard methods described above. The changes in the dCA indexes can then be tracked from the parameters obtained according to the analysis method used (see above). There are two choices to be made in the sliding window: the length and the shape of the window function that are used. The length of this sliding window leads to a compromise between time and frequency resolution, since high temporal resolution is achieved only with low frequency resolution and vice versa; shorter windows also lead to reduced statistical robustness. A tapered window allows more weight to be allocated to (usually) the centre of the data window under analysis. Wavelets give an alternative decomposition with temporal resolution adapted frequency by frequency; however this is more complicated to implement. The available non-stationary techniques that have been used in the context of cerebral autoregulation have been summarised recently:^62^ these methods include ARMA models with sliding windows, recursive least-squares, Laguerre-Volterra networks, wavelet phase synchronization and multimodal analysis. The non-stationarity of cerebral autoregulation, as measured using phase shift, has been clearly demonstrated.^47^

Time-varying filters have been used in the context of cerebral autoregulation.^63^ The first method was based on the Wigner-Ville distribution to calculate an instantaneous transfer function, whereas the second and third methods were based on an adaptive filter and an ensemble Kalman filter respectively. Repeated application of least squares methods have been used to derive the coefficients of linear (infinite impulse response – autoregressive moving average) filters, based on windowed sections of data.^38,66^

Wavelet phase synchronization was proposed by Latka et al.^64^ to quantify the variability in phase angle between BP and CBFV. Once wavelets have been used to calculate the instantaneous phase angle, synchronization is calculated: the value of this parameter lies between 0 and 1, where 0 represents a uniform random distribution over time and 1 a constant value over time. The use of wavelets means that this parameter can be calculated as a function of scale/frequency. In the very low frequency (high scale) range, this parameter is found to be very low, which can be taken as a measure of intact autoregulation (although care always has to be taken in interpreting any finding as a direct measure of autoregulation).

Finally, we mention the use of multimodal analysis, which is based on the use of empirical mode decomposition to break down the signal into intrinsic mode functions.^65^ Although each mode is oscillatory, they can have time-varying amplitude and frequency, which allows them to map out non-stationarities in the data. Through the use of the Hilbert transform, the instantaneous phase of each mode can be calculated. The comparison between normal subjects and subjects with stroke or hypertension has shown greater repeatability in phase angle than in ARI,^12,66^ indicating the value of this technique. On the other hand, the selection of which mode functions to use is rather subjective, which is a considerable limitation for the diffusion and standardization of this approach.

Time-variant methods have thus shown some interesting preliminary results, although the number of available techniques means that choosing a method can be difficult, with each method involving a number of choices of parameter values for a practical implementation. The temporal variability that has been shown by the available studies also remains to be quantified properly and its physiological relevance identified. As concluded by Panerai,^60^ “one key priority for future work is the development and validation of multivariate time-varying techniques to minimise the influence of the many co-variates which contribute to … non-stationarity”.

## Future perspectives, challenges and pitfalls

The previous papers in this series of reviews on autoregulation in the study of stroke have clearly shown the promise, but also the challenges in assessing CA. There are very many different methods for assessing CA in use, involving differences in the techniques for measuring the physiological signals, a range of protocols and finally many options for the calculation of indexes to quantify CA. This section will discuss some of these challenges and identify key areas requiring further research, focussing on dCA.

We begin with measurement methods, where we are still limited by what we can measure with current non-invasive methods. However it is also not immediately obvious what we would like to measure, even if we could: flow (not just velocity) in major arteries, or perfusion of neuronal beds, oxygen supply or uptake; how to measure (and define) perfusion pressure; should we aim for local dCA in specific vascular beds (which may miss impairment in regions not measured) or global dCA (which may miss localized impairment in focal strokes^67^) It has become customary to work with mean (beat-averaged) CBF and BP signals, but there is some indication that systolic values could provide additional benefit.^8,68,69^

A number of recent studies have addressed the time-varying behaviour of cerebral autoregulation,^12,25,36,45–47,60,66,67,73^ with suggestions that some of the variability is physiological change, in addition to ‘noise’ in the data and consequent random errors in dCA estimates. A recent consensus statement on management of severe traumatic brain injury^74^ has recognised the importance of continuous CA monitoring and time-varying approaches might present an opportunity for timely MAP adjustments to achieve more efficient and personalised care. There is also evidence of inter-individual differences, even in healthy volunteers, given the significant values of intra-class correlation coefficients (ICC).^75,76^

The length of recording required has been under discussion for some time. Probably the most common duration for dCA assessment is between 5 and 10 minutes,^70,77^ with Claassen, Meel-van den Abeelen^10^ recommending a minimum of 5 minutes for TFA from spontaneous variations in BP and CBFV. The original work on the Mx parameter used two hours of recording in each subject,^8^ and the calculation of the Mx over successive 3 minute intervals, but 5 to 10 minute intervals seem now more common,^36,70,77^ with recordings typically upward of 20 minutes in duration; this allows either averaging to obtain a more robust estimate, or tracking of time-varying CA. The work both of Chi, Wang^77^ and Mahdi, Rutter^78^ has suggested that 5–10 minutes is an appropriate duration of recording for individual estimates.

It has been repeatedly reported that the agreement between different dCA indexes calculated from a cohort of individuals is not always strong (and sometimes absent),^36,48,49^ in spite of good evidence that the different methods all reflect autoregulatory behaviours. One interpretation of this result is that different methods provide complementary information about the complex autoregulatory process. However, results may also be confounded when correlations are calculated using healthy individuals only, where the range of dCA indices is narrow, such that any random errors can rapidly reduce correlations.

Perhaps the biggest challenge we have is ‘bootstrapping’ our field to obtain a gold-standard method to assess dCA. This involves moving from a concept of autoregulation to a method that can robustly quantify the construct in a physiologically and clinically meaningful manner. If we had a gold-standard (even one that perhaps was not suitable for routine use in the clinic or in research), it would be much easier to identify an optimized approximation. Until we have such a gold standard, the many reasonable options for methods that are currently used to quantify CA add to the difficulty of developing our field, because results from different studies often cannot be readily compared. We also have many possible criteria for comparing alternative approaches. These include the methods’ ability to predict outcomes for patients or to classify patients/subjects according to whether CA impairment might be expected (e.g. by clinical condition or after inhalation of CO_2_ which is known to temporarily impair CA). Furthermore, consistency in repeated measurements (repeatability), statistical robustness of estimates, consistency within a ‘similar’ cohort, or agreement with alternative (but sadly not gold-standard) measures provide alternative criteria in selecting CA measures (e.g. CARNet’s multicentre studies^50,79,80^). How well a measure reflects our conceptual understanding of autoregulation has probably been the primary guide in the initial (and probably often final) choice of preferred approaches. The simplicity of methods, and the ability to explain the concepts that underpin the calculations, are further factors in preferring some approaches over others.

Finally, we would like to indicate some priorities for further research into dCA analysis.

There is currently no consensus on the ‘best’ method (measurement, protocol and signal analysis) for the study of stroke, and little evidence to indicate which method should be broadly adopted. Such a consensus would greatly facilitate linking studies between groups to build up a more comprehensive picture of CA impairment in stroke. There is promise that such a consensus is possible, based on previous work on TFA,^10^ though this focussed only on this one method without evidence that it was ‘optimal’ in the study of stroke. CARNet (www.car-net.org) is currently in the early stages of developing such a consensus.

Further research priorities are:

- Improve understanding of specific impairments in CA following stroke. Given the complexity of the mechanisms and different vascular territories that might be affected, the sometimes limited agreement between analysis techniques might reflect different aspects (features) of autoregulation that can be affected in different cases. It may be that good/impaired autoregulation cannot be quantified by a single index, but requires multidimensional analysis, which only some of these dimensions (indexes of dCA) being affected in specific clinical conditions.
- Disentangle the effect of dCA from those of related physiological control systems, including neurovascular coupling, cerebrovascular reactivity and baroreflex.
- Assess whether dCA estimated from small fluctuations in BP and CBFV is a good predictor of behaviours during large (and clinically more significant) fluctuations.
- Improve understanding of time-varying behaviour of cerebral autoregulation and the potential benefits of continuous monitoring of dCA in the acute and sub-acute phase following stroke.
- Identify which co-variates (pCO_2_, metabolic activity etc.) need to be included in the estimation of dCA or whether we need to take these into account in some other way.
- On a more practical side, methods for (semi)automated editing of recorded signals are highly desirable, which would greatly reduce the time required for dCA analysis and facilitate automated, bedside technology for continuous patient monitoring. This is particularly desirable in patient groups where it is difficult to record extended signals of high quality.
- Provide not only an estimate for the dCA index, but also confidence limits, or some other indication of whether in a particular recording, the estimate is valid.

We look forward to new developments and further progress in this challenging, exciting and evolving field and achieving the benefits for patients that we are all seeking.

## References

[1] Paulson OB Strandgaard S Edvinsson L. Cerebral autoregulation. Cerebrovasc Brain Metab Rev 1990; 2: 161–192. 2201348

[2] Panerai RB. Assessment of cerebral pressure autoregulation in humans – a review of measurement methods. Physiol Meas 1998; 19: 305–338. 9735883 10.1088/0967-3334/19/3/001

[3] Intharakham K Beishon L Panerai RB , et al. Assessment of cerebral autoregulation in stroke: a systematic review and meta-analysis of studies at rest. J Cereb Blood Flow Metab 2019; 39: 2105–2116.. 31433714 10.1177/0271678X19871013 PMC6827119

[4] Czosnyka M Czosnyka Z Smielewski P. Pressure reactivity index: journey through the past 20 years. Acta Neurochir (Wien) 2017; 159: 2063–2065. 28849287 10.1007/s00701-017-3310-1

[5] Zweifel C Castellani G Czosnyka M , et al. Continuous assessment of cerebral autoregulation with near-infrared spectroscopy in adults after subarachnoid hemorrhage. Stroke 2010; 41: 1963–1968. 20651272 10.1161/STROKEAHA.109.577320

[6] Petkus V Preiksaitis A Krakauskaite S , et al. Non-invasive cerebrovascular autoregulation assessment using the volumetric reactivity index: prospective study. Neurocrit Care 2019; 30: 42–50. 29951960 10.1007/s12028-018-0569-x

[7] Yang M Yang Z Yuan T , et al. A systemic review of functional near-Infrared spectroscopy for stroke: current application and future directions. Front Neurol 2019; 10: 58–02. 10.3389/fneur.2019.00058 PMC6371039 30804877

[8] Czosnyka M Smielewski P Kirkpatrick P , et al. Monitoring of cerebral autoregulation in head-injured patients. Stroke 1996; 27: 1829–1834. 8841340 10.1161/01.str.27.10.1829

[9] Panerai RB Eames PJ Potter JF. Multiple coherence of cerebral blood flow velocity in humans. Am J Physiol Heart Circ Physiol 2006; 291: H251–259. 16489099 10.1152/ajpheart.01348.2005

[10] Claassen JAHR Meel-van den Abeelen ASS Simpson DM , et al.; International Cerebral Autoregulation Research Network (CARNet). Transfer function analysis of dynamic cerebral autoregulation: a white paper from the international cerebral autoregulation research network. J Cereb Blood Flow Metab 2016; 36: 665–680. 26782760 10.1177/0271678X15626425 PMC4821028

[11] Mitsis GD Debert CT Hajo MI , et al. Nonlinear, multiple-input modeling of cerebral hemodynamics during baseline and hypercapnia in young and post-menopausal women. Annu Int Conf IEEE Eng Med Biol Soc 2007; 2007: 2855–2858. 18002590 10.1109/IEMBS.2007.4352924

[12] Novak V Yang AC Lepicovsky L , et al. Multimodal pressure-flow method to assess dynamics of cerebral autoregulation in stroke and hypertension. Biomed Eng Online 2004; 3: 39. 15504235 10.1186/1475-925X-3-39 PMC529459

[13] Zeiler FA Donnelly J Calviello L , et al. Pressure autoregulation measurement techniques in adult traumatic brain injury, part II: a scoping review of continuous methods. J Neurotrauma 2017; 34: 3224–3237. 28699412 10.1089/neu.2017.5086

[14] Zeiler FA Ercole A Czosnyka M , et al. Continuous cerebrovascular reactivity monitoring in moderate/severe traumatic brain injury: a narrative review of advances in neurocritical care. Br J Anaesth 2020; 124: 440–453. 10.1016/j.bja.2019.11.031 31983411

[15] Cipolla M. The cerebral circulation. San Rafael, CA: Morgan & Claypool Life Sciences, 2009. 21452434

[16] Payne S. Cerebral autoregulation: control of blood flow in the brain. Switzerland: Springer, 2016.

[17] Xiong L Liu XY Shang T , et al. Impaired cerebral autoregulation: measurement and application to stroke. J Neurol Neurosurg Psychiatry 2017; 88: 520–531. 28536207 10.1136/jnnp-2016-314385

[18] Tzeng YC Panerai RB. CrossTalk proposal: dynamic cerebral autoregulation should be quantified using spontaneous blood pressure fluctuations. J Physiol 2018; 596: 3–5. 29207213 10.1113/JP273899 PMC5746519

[19] Simpson D Claassen J. CrossTalk opposing view: dynamic cerebral autoregulation should be quantified using induced (rather than spontaneous) blood pressure fluctuations. J Physiol 2018; 596: 7–9. 29207208 10.1113/JP273900 PMC5746528

[20] Panerai RB Kelsall AWR Rennie JM , et al. Cerebral autoregulation dynamics in premature newborns. Stroke 1995; 26: 74–80. 7839402 10.1161/01.str.26.1.74

[21] Numan T Bain AR Hoiland RL , et al. Static autoregulation in humans: a review and reanalysis. Med Eng Phys 2014; 36: 1487–1495. 25205587 10.1016/j.medengphy.2014.08.001

[22] Tiecks FP Lam AM Aaslid R , et al. Comparison of static and dynamic cerebral autoregulation measurements. Stroke 1995; 26: 1014–1019. 7762016 10.1161/01.str.26.6.1014

[23] Ramaekers VT Casaer P Daniels H , et al. The influence of blood transfusion on brain blood flow autoregulation among stable preterm infants. Early Hum Dev 1992; 30: 211–220. 1468384 10.1016/0378-3782(92)90070-w

[24] Aaslid R Lindegaard KF Sorteberg W , et al. Cerebral autoregulation dynamics in humans. Stroke 1989; 20: 45–52. 2492126 10.1161/01.str.20.1.45

[25] Panerai RB Saeed NP Robinson TG. Cerebrovascular effects of the thigh cuff maneuver. Am J Physiol Heart Circ Physiol 2015; 308: H688–H696. 25659488 10.1152/ajpheart.00887.2014 PMC4385993

[26] Marmarelis VZ Mitsis GD Shin DC. Multiple-input nonlinear modelling of cerebral hemodynamics using spontaneous arterial blood pressure, end-tidal CO2 and heart rate measurements. Philos T R Soc A 2016; 374: 20150180. 10.1098/rsta.2015.0180 PMC4822442 27044989

[27] Panerai RB Moody M Eames PJ , et al. Cerebral blood flow velocity during mental activation: interpretation with different models of the passive pressure-velocity relationship. J Appl Physiol (1985) 2005; 99: 2352–2362. 16099892 10.1152/japplphysiol.00631.2005

[28] Deegan BM Serrador JM Nakagawa K , et al. The effect of blood pressure calibrations and transcranial doppler signal loss on transfer function estimates of cerebral autoregulation. Med Eng Phys 2011; 33: 553–562. 21239208 10.1016/j.medengphy.2010.12.007 PMC4394242

[29] Eames PJ Potter JF Panerai RB. Assessment of cerebral autoregulation from ectopic heartbeats. Clin Sci (Lond) 2005; 109: 109–115. 15773816 10.1042/CS20050009

[30] Gommer ED Shijaku E Mess WH , et al. Dynamic cerebral autoregulation: different signal processing methods without influence on results and reproducibility. Med Biol Eng Comput 2010; 48: 1243–1250. 21049290 10.1007/s11517-010-0706-y PMC2993898

[31] Panerai RB Robinson TG Minhas JS. The upper frequency limit of dynamic cerebral autoregulation. J Physiol 2019; 597: 5821–5833. 31671473 10.1113/JP278710

[32] Wagner EM Traystman RJ. Cerebrovascular transmural pressure and autoregulation. Ann Biomed Eng 1985; 13: 311–320. 4037460 10.1007/BF02584249

[33] Panerai RB. The critical closing pressure of the cerebral circulation. Med Eng Phys 2003; 25: 621–632. 12900178 10.1016/s1350-4533(03)00027-4

[34] Sorrentino E Budohoski KP Kasprowicz M , et al. Critical thresholds for transcranial doppler indices of cerebral autoregulation in traumatic brain injury. Neurocrit Care 2011; 14: 188–193. 21181299 10.1007/s12028-010-9492-5

[35] Lewis PM Smielewski P Pickard JD , et al. Dynamic cerebral autoregulation: should intracranial pressure be taken into account? Acta Neurochir (Wien) 2007; 149: 549–555; discussion 555. 17476455 10.1007/s00701-007-1160-y

[36] Liu X Czosnyka M Donnelly J , et al. Comparison of frequency and time domain methods of assessment of cerebral autoregulation in traumatic brain injury. J Cereb Blood Flow Metab 2015; 35: 248–256. 25407266 10.1038/jcbfm.2014.192 PMC4426741

[37] Panerai RB Haunton VJ Llwyd O , et al. Cerebral critical closing pressure and resistance-area product: the influence of dynamic cerebral autoregulation, age, and sex. *J Cereb Blood Flow Metab*. Epub ahead of print 4 April 2021. DOI: 10.1177/0271678X211004131. 10.1177/0271678X211004131 PMC8392773 33818187

[38] Panerai RB Eames PJ Potter JF. Variability of time-domain indices of dynamic cerebral autoregulation. Physiol Meas 2003; 24: 367–381. 12812422 10.1088/0967-3334/24/2/312

[39] Liu Y Allen R. Analysis of dynamic cerebral autoregulation using an ARX model based on arterial blood pressure and middle cerebral artery velocity simulation. Med Biol Eng Comput 2002; 40: 600–605. 12452423 10.1007/BF02345461

[40] Panerai RB Rennie JM Kelsall AWR , et al. Frequency-domain analysis of cerebral autoregulation from spontaneous fluctuations in arterial blood pressure. Med Biol Eng Comput 1998; 36: 315–322. 9747571 10.1007/BF02522477

[41] Zhang R Zuckerman JH Giller CA , et al. Transfer function analysis of dynamic cerebral autoregulation in humans. Am J Physiol-Heart C 1998; 274: H233–H241. 10.1152/ajpheart.1998.274.1.h233 9458872

[42] Jachan M Reinhard M Spindeler L , et al. Parametric versus nonparametric transfer function estimation of cerebral autoregulation from spontaneous blood-pressure oscillations. Cardiovasc Eng 2009; 9: 72–82. 19475507 10.1007/s10558-009-9072-5

[43] Angarita-Jaimes N Kouchakpour H Liu J , et al. Optimising the assessment of cerebral autoregulation from black box models. Med Eng Phys 2014; 36: 607–612. 24508528 10.1016/j.medengphy.2013.12.012

[44] Giller CA. The frequency-dependent behavior of cerebral autoregulation. Neurosurgery 1990; 27: 362–368. 2234328 10.1097/00006123-199009000-00004

[45] Tian F Tarumi T Liu H , et al. Wavelet coherence analysis of dynamic cerebral autoregulation in neonatal hypoxic–ischemic encephalopathy. NeuroImage: Clin 2016; 11: 124–132. 26937380 10.1016/j.nicl.2016.01.020 PMC4753811

[46] Chalak LF Zhang R. New wavelet neurovascular bundle for bedside evaluation of cerebral autoregulation and neurovascular coupling in newborns with hypoxic-ischemic encephalopathy. Dev Neurosci 2017; 39: 89–96. 28355608 10.1159/000457833 PMC5519424

[47] Placek MM Wachel P Iskander DR , et al. Applying time-frequency analysis to assess cerebral autoregulation during hypercapnia. Plos One 2017; 12: e0181851. 28750024 10.1371/journal.pone.0181851 PMC5531479

[48] Tzeng YC Ainslie PN Cooke WH , et al. Assessment of cerebral autoregulation: the quandary of quantification. Am J Physiol-Heart C 2012; 303: H658–H671. 10.1152/ajpheart.00328.2012 22821992

[49] Czosnyka M Smielewski P Lavinio A , et al. An assessment of dynamic autoregulation from spontaneous fluctuations of cerebral blood flow velocity: a comparison of two models, index of autoregulation and mean flow index. Anesth Analg 2008; 106: 234–239, table of contents. 18165583 10.1213/01.ane.0000295802.89962.13

[50] Meel-van den Abeelen ASS Simpson DM Wang LJY , et al. Between-centre variability in transfer function analysis, a widely used method for linear quantification of the dynamic pressure-flow relation: the CARNet study. Med Eng Phys 2014; 36: 620–627. 24725709 10.1016/j.medengphy.2014.02.002 PMC4155942

[51] Panerai RB Moody M Eames PJ , et al. Dynamic cerebral autoregulation during brain activation paradigms. Am J Physiol Heart Circ Physiol 2005; 289: H1202–1208. 15863461 10.1152/ajpheart.00115.2005

[52] Panerai RB Haunton VJ Hanby MF , et al. Statistical criteria for estimation of the cerebral autoregulation index (ARI) at rest. Physiol Meas 2016; 37: 661–672. 27093173 10.1088/0967-3334/37/5/661

[53] Panerai RB Deverson ST Mahony P , et al. Effects of CO2 on dynamic cerebral autoregulation measurement. Physiol Meas 1999; 20: 265–275. 10475580 10.1088/0967-3334/20/3/304

[54] Katsogridakis E Simpson DM Bush G , et al. Revisiting the frequency domain: the multiple and partial coherence of cerebral blood flow velocity in the assessment of dynamic cerebral autoregulation. Physiol Meas 2016; 37: 1056–1073. 27244196 10.1088/0967-3334/37/7/1056

[55] Peng T Rowley AB Ainslie PN , et al. Multivariate system identification for cerebral autoregulation. Ann Biomed Eng 2008; 36: 308–320. 18066666 10.1007/s10439-007-9412-9

[56] Panerai RB Dawson SL Potter JF. Linear and nonlinear analysis of human dynamic cerebral autoregulation. Am J Physiol 1999; 277: H1089–1099. 10484432 10.1152/ajpheart.1999.277.3.H1089

[57] Mitsis GD Zhang R Levine BD , et al. Modeling of nonlinear physiological systems with fast and slow dynamics. II. Application to cerebral autoregulation. Ann Biomed Eng 2002; 30: 555–565. 12086006 10.1114/1.1477448

[58] Panerai RB Chacon M Pereira R , et al. Neural network modelling of dynamic cerebral autoregulation: assessment and comparison with established methods. Med Eng Phys 2004; 26: 43–52. 14644597 10.1016/j.medengphy.2003.08.001

[59] Chacon M Araya C Panerai RB. Non-linear multivariate modeling of cerebral hemodynamics with autoregressive support vector machines. Med Eng Phys 2011; 33: 180–187. 21051271 10.1016/j.medengphy.2010.09.023

[60] Bellapart J and Fraser JF. Transcranial Doppler assessment of cerebral autoregulation. *Ultrasound Med Biol* 2009; 35: 883–893. 2009/03/31. DOI: 10.1016/j.ultrasmedbio.2009.01.005. 10.1016/j.ultrasmedbio.2009.01.005 19329245

[61] Müller M. Giller CA, Müller M: Linearity and nonlinearity in cerebral hemodynamics. *Med Eng Phys* 2003; 25: 633–646. doi: 10.1016/s1350-4533(03)00028-6. 10.1016/s1350-4533(03)00028-6 12900179

[62] Panerai RB. Nonstationarity of dynamic cerebral autoregulation. *Med Eng Phys* 2014; 36: 576–584. 10.1016/j.medengphy.2013.09.004 24113077

[63] Aoi MC, Matzuka BJ and Olufsen MS. Toward online, noninvasive, nonlinear assessment of cerebral autoregulation. *Conf Proc IEEE Eng Med Biol Soc* 2011; 2011: 2410–2413. 2012/01/19. DOI: 10.1109/IEMBS.2011.6090671 10.1109/IEMBS.2011.6090671 22254827

[64] Noack F Christ M May SA , et al. Assessment of dynamic changes in cerebral autoregulation. Biomed Tech (Berl) 2007; 52: 31–36. 17313331 10.1515/BMT.2007.007

[65] Liu J Simpson MD Yan JY , et al. Tracking time-varying cerebral autoregulation in response to changes in respiratory PaCO2. Physiol Meas 2010; 31: 1291–1307. 20720290 10.1088/0967-3334/31/10/001

[66] Liu J Simpson DM Kouchakpour H , et al. Rapid pressure-to-flow dynamics of cerebral autoregulation induced by instantaneous changes of arterial CO2. Med Eng Phys 2014; 36: 1636–1643. 25287624 10.1016/j.medengphy.2014.09.005

[67] Latka M Turalska M Glaubic-Latka M , et al. Phase dynamics in cerebral autoregulation. Am J Physiol Heart Circ Physiol 2005; 289: H2272–2279. 16024579 10.1152/ajpheart.01307.2004

[68] Huang NE Shen Z Long SR , et al. The empirical mode decomposition and the Hilbert spectrum for nonlinear and non-stationary time series analysis. Proc R Soc Lond A 1998; 454: 903–995.

[69] Hu K Peng CK Czosnyka M , et al. Nonlinear assessment of cerebral autoregulation from spontaneous blood pressure and cerebral blood flow fluctuations. Cardiovasc Eng 2008; 8: 60–71. 18080758 10.1007/s10558-007-9045-5 PMC2765466

[70] Reinhard M Rutsch S Hetzel A. Cerebral autoregulation in acute ischemic stroke. Perspect Med 2012; 1: 194–197.

[71] Rosengarten B Kaps M. Peak systolic velocity doppler index reflects most appropriately the dynamic time course of intact cerebral autoregulation. Cerebrovasc Dis 2002; 13: 230–234. 12011546 10.1159/000057848

[72] Smirl JD Wright AD Ainslie PN , et al. Differential systolic and diastolic regulation of the cerebral pressure-flow relationship during squat-stand manoeuvres. Acta Neurochir Suppl 2018; 126: 263–268. 29492572 10.1007/978-3-319-65798-1_52

[73] Addison PS. A review of wavelet transform time-frequency methods for NIRS-Based analysis of cerebral autoregulation. IEEE Rev Biomed Eng 2015; 8: 78–85. 26011892 10.1109/RBME.2015.2436978

[74] Hawryluk GWJ Aguilera S Buki A , et al. A management algorithm for patients with intracranial pressure monitoring: the Seattle international severe traumatic brain injury consensus conference (SIBICC). Intensive Care Med 2019; 45: 1783–1794. 31659383 10.1007/s00134-019-05805-9 PMC6863785

[75] Ortega-Gutierrez S Petersen N Masurkar A , et al. Reliability, asymmetry, and age influence on dynamic cerebral autoregulation measured by spontaneous fluctuations of blood pressure and cerebral blood flow velocities in healthy individuals. J Neuroimaging 2014; 24: 379–386. 23607680 10.1111/jon.12019 PMC4812577

[76] Brodie FG Atkins ER Robinson TG , et al. Reliability of dynamic cerebral autoregulation measurement using spontaneous fluctuations in blood pressure. Clin Sci 2009; 116: 513–520. 10.1042/CS20080236 18939945

[77] Chi N-F Wang C-Y Chan L , et al. Comparing different recording lengths of dynamic cerebral autoregulation: 5 versus 10 minutes. BioMed Res Int 2018; 2018: 7803426. 29662898 10.1155/2018/7803426 PMC5831790

[78] Mahdi A Rutter EM Payne SJ. Effects of non-physiological blood pressure artefacts on cerebral autoregulation. Med Eng Phys 2017; 47: 218–221. 28694107 10.1016/j.medengphy.2017.06.007

[79] Sanders ML Claassen JAHR Aries M , et al. Reproducibility of dynamic cerebral autoregulation parameters: a multi-centre, multi-method study. Physiol Meas 2018; 39: 125002. 30523976 10.1088/1361-6579/aae9fd

[80] Elting JW Sanders ML Panerai RB , et al. Assessment of dynamic cerebral autoregulation in humans: is reproducibility dependent on blood pressure variability? Plos One 2020; 15: e0227651. 31923919 10.1371/journal.pone.0227651 PMC6954074

[81] Fan LK Bush G Katsogridakis E , et al. Adaptive feedback analysis and control of programmable stimuli for assessment of cerebrovascular function. Med Biol Eng Comput 2013; 51: 709–718. 23389239 10.1007/s11517-013-1040-y

[82] Katsogridakis E Bush G Fan LK , et al. Detection of impaired cerebral autoregulation improves by increasing arterial blood pressure variability. J Cereb Blood Flow Metab 2013; 33: 519–523. 23232946 10.1038/jcbfm.2012.191 PMC3618385

[83] Serrador JM Sorond FA Vyas M , et al. Cerebral pressure-flow relations in hypertensive elderly humans: transfer gain in different frequency domains. J Appl Physiol (1985) 2005; 98: 151–159. 15361517 10.1152/japplphysiol.00471.2004

[84] Panerai RB Dawson SL Eames PJ , et al. Cerebral blood flow velocity response to induced and spontaneous sudden changes in arterial blood pressure. Am J Physiol-Heart C 2001; 280: H2162–H2174. 10.1152/ajpheart.2001.280.5.H2162 11299218

[85] Birch AA Neil-Dwyer G Murrills AJ. The repeatability of cerebral autoregulation assessment using sinusoidal lower body negative pressure. Physiol Meas 2002; 23: 73–83. 11876243 10.1088/0967-3334/23/1/307

[86] Birch AA Dirnhuber MJ Hartley-Davies R , et al. Assessment of autoregulation by means of periodic changes in blood pressure. Stroke 1995; 26: 834–837. 7740576 10.1161/01.str.26.5.834

